# Health care seeking behaviour towards cervical cancer screening among women aged 30–49 years in Arbaminch town, Southern Ethiopia, 2023

**DOI:** 10.1186/s12885-023-11810-5

**Published:** 2024-01-05

**Authors:** Begetayinoral Kussia, Misgun Shewangizaw, Samuel Abebe, Habtamu Alemu, Tesfahun Simon

**Affiliations:** 1 Department of Midwifery, College of Medicine and Health Science, Arbaminch University, Arbaminch, P.O. Box 21, Ethiopia; 2 School of public health, College of Medicine and Health Science, Arbaminch University, Arbaminch, P.O. Box 21, Ethiopia

**Keywords:** Cervical cancer, Health care seeking behavior and health belief model

## Abstract

**Background:**

Cervical cancer is a preventable disease. However, it remains the commonest and deadly cancer in women worldwide. Health care seeking behaviour is not well studied in Ethiopia even though it is crucial in averting cervical cancer by maximizing cervical cancer screening utilization. Therefore, this study amid to assess health care seeking behaviour towards cervical cancer screening and its associated factors among women aged 30–49 years in Arba Minch town, Southern Ethiopia, 2023.

**Methods:**

A community-based cross-sectional study design was conducted on 414 women who are in the age range of 30–49 in Arba Minch town from January 2-February20, 2023. Study participants were selected by a simple random sampling technique from all kebeles and data were collected using pretested interviewer administered questionnaires. SPSS version 27 was used to conduct binary and multivariable logistic regression analysis. Socio-demographic characteristics of the respondents were described using descriptive statistics. Furthermore, binary and multivariable logistic regression analyses were made to find the factors associated with health care seeking behaviour. Variables with a *p*-value less than 0.25 on binary logistic regression were selected for multivariable logistic regression. Variables with a *p*-value < 0.05 were considered statistically significant. The reliability and internal consistency of the constructs of health belief model were calculated independently using Cronbach’s alpha.

**Result:**

The prevalence of health care seeking behaviour towards cervical cancer screening was 197(47.6%) [95%CI: 42.7-52.5%]. Respondents’ good knowledge [AOR = 1.55, 95%CI: 1.01–2.39], positive perceived susceptibility [AOR = 3.63, 95%CI: 2.06–6.42], positive perceived severity [AOR = 2.65, 95%CI: 1.71–4.09], positive perceived benefits [AOR = 4.85, 95%CI: 2.92–7.87] were significantly associated with health seeking behaviour.

**Conclusion:**

The prevalence of health care seeking behaviour towards cervical cancer screening is low in this study. To maximize the health care seeking behavior of women, further acting on perceived susceptibility, respondents’ knowledge, perceived severity, and perceived benefit of the woman are crucial.

## Background

Globally, cervical cancer is the fourth most common cancer in women with an expected 604, 000 new occurrences and 342, 000 deaths in the year 2020 [1].. In Africa, cervical cancer is the leading cause of cancer death amongst women, with an estimated 117,316 new cervical cancer cases each year, making cervical cancer the 2nd leading cause of female cancer and the 2nd most common female cancer among women aged 15 to 44 years in the continent [2]. Africa carries the greatest burden, with 24.55% of the global mortality from cervical cancer [3]. Eastern Africa alone shares the highest burden with an estimated 54,560 annual number of new cervical cancer cases [2]. 

According to Information Centre on HPV and Cancer (estimation for 2020), about 7,445 new cervical cancer cases and 5335 deaths occur each year in Ethiopia, making cervical cancer the 2nd top cause of female cancer in the country [4].

Human Papilloma Virus (HPV), sexual history, smoking, Chlamydia infection, birth control pills, having multiple full-term pregnancies, young age at first full-term pregnancy and having a weakened immune system are some of the identified risk factors of cervical cancer [5]. Among the risk factors listed majority of cervical cancer is caused by HPV. Currently, around 13 different types of HPV have been identified based on their potential to be cancerous [6]. Among high grade cervical pre-cancers, HPV16 and 18 are responsible for nearly 70% of cervical cancer cases [2].

**Fig. 1:**
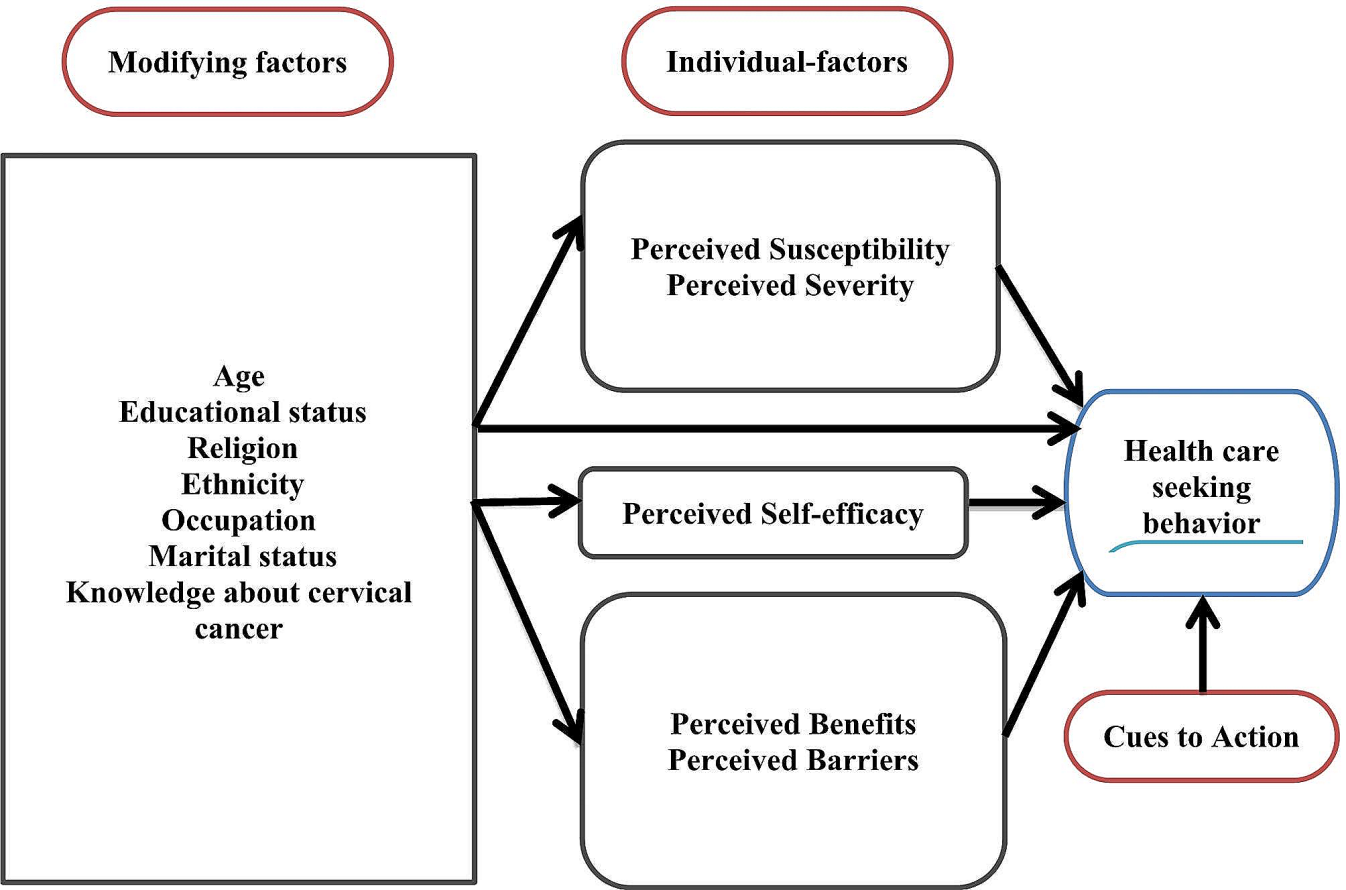
Diagram Illustrating the health belief model [11]

Health care seeking behavior toward cervical cancer gives a chance for the application of both primary and secondary prevention strategies for wide-ranging prevention and control strategies of cervical cancer [7]. The Health Belief Model (HBM) emphases on the causes of health-related behaviors, with factors consisting of perceived susceptibility and severity of a health problem, perceived benefits and barriers of conducting health-related behaviors, cues to action, and other socio-demographic factors (Fig. 1) [8]. According to the HBM, women are more likely to engage in health care seeking behavior if their perceptions of susceptibility and seriousness are high, the barriers to do such behaviors are low, and the benefits of engaging in such health behaviors are substantial [9]. According to previous studies, factors associated with poor health care seeking behavior were poor knowledge, not ever received information, education level of the respondents, and not actively searching information [7, 10].

According to the literature, factors associated with poor health care seeking behavior were poor knowledge, not ever received information, education level of the respondents, and not actively searching information [7, 10].

To significantly drop the incidence and mortality caused by cervical cancer, by maximizing the cervical cancer screening service utilization, such barriers must be addressed. Thus, awareness should be created, and there must be effective screening and prevention services that facilitate early detection and treatment. To address this issue, Behavioral change communication (BCC) is one strategy used to impact social norms, promote behaviour change, and raise awareness of cervical cancer prevention in a particular group of people or subpopulations according to Federal Democratic Republic of Ethiopia Ministry of Health guideline for cervical cancer prevention and control [12, 13]. There is insufficient data on health care seeking behaviour for the prevention and control of cervical cancer in Ethiopia according to what is specified in the national strategic Plan for the prevention and control of chronic diseases [13]. Furthermore, there are no community based studies conducted so far on health care seeking behaviour towards cervical cancer screening among women aged 30–49 years in the country. Therefore, this study aims to assess health seeking behavior towards cervical cancer screening and associated factors among women aged 30–49 years using the HBM as a guiding theoretical framework in Arba Minch town, Southern Ethiopia, 2023.

## Methods

### Study area and period

The study was conducted in Arba Minch town, which is located about 495 km south of Addis Ababa, the capital city of Ethiopia, and 275 km from Hawassa, the capital of Southern Nations and Nationalities People region. The total number of population of Arba Minch town in the year 2014E.C is 123,446 of which around 61,970 (50.2%) are females. The number of women in the age range of 30–49 in the town is 11,898. There are twelve kebeles (the smallest administrative units in the country) in the town and there are a total of 4 health facilities (1 general hospital, 1 primary hospital, and two health centers). The data was collected from January 2-February20, 2023.

### Study design

A community-based cross-sectional study design was conducted.

### Study participants

All 30–49 years women living in Arba Minch town were the source population and women who had the chance of being randomly selected from the source population at a household level were the study population. All 30–49 year women (including pregnant women) who had lived in the study area for at least 6 months were included in the current study whereas, women who were already screened and diagnosed for cervical cancer and those women who have had total hysterectomy were excluded from this study.

### Sampling

#### Sample size

The sample size in the current study was calculated using a single population proportion formula, with an assumption of margin of error 5%, 95% confidence level, and we used a 50% proportion of health care seeking behavior since there is no previous study on the topic among women aged 30–49 years.

$$N\, = \,(Z{\bf{\alpha }}/{\bf{2}}){\bf{2}}\,{\bf{p}}\,\left( {{\bf{1}} - {\bf{p}}} \right)/{\bf{d2}}$$

Where N = desired sample size, *p* = assumed proportion of health seeking behavior.

Z (α/2) = critical value at 95% confidence level of certainty (1.96) and.

d = margin of error between the sample and population = 5%.

*n* = (𝑍α/2)2 *p* (1-*p*)/d2 = 384 considering 10% none response rate, the final sample size was 422.

#### Sampling techniques

**Fig. 2:**
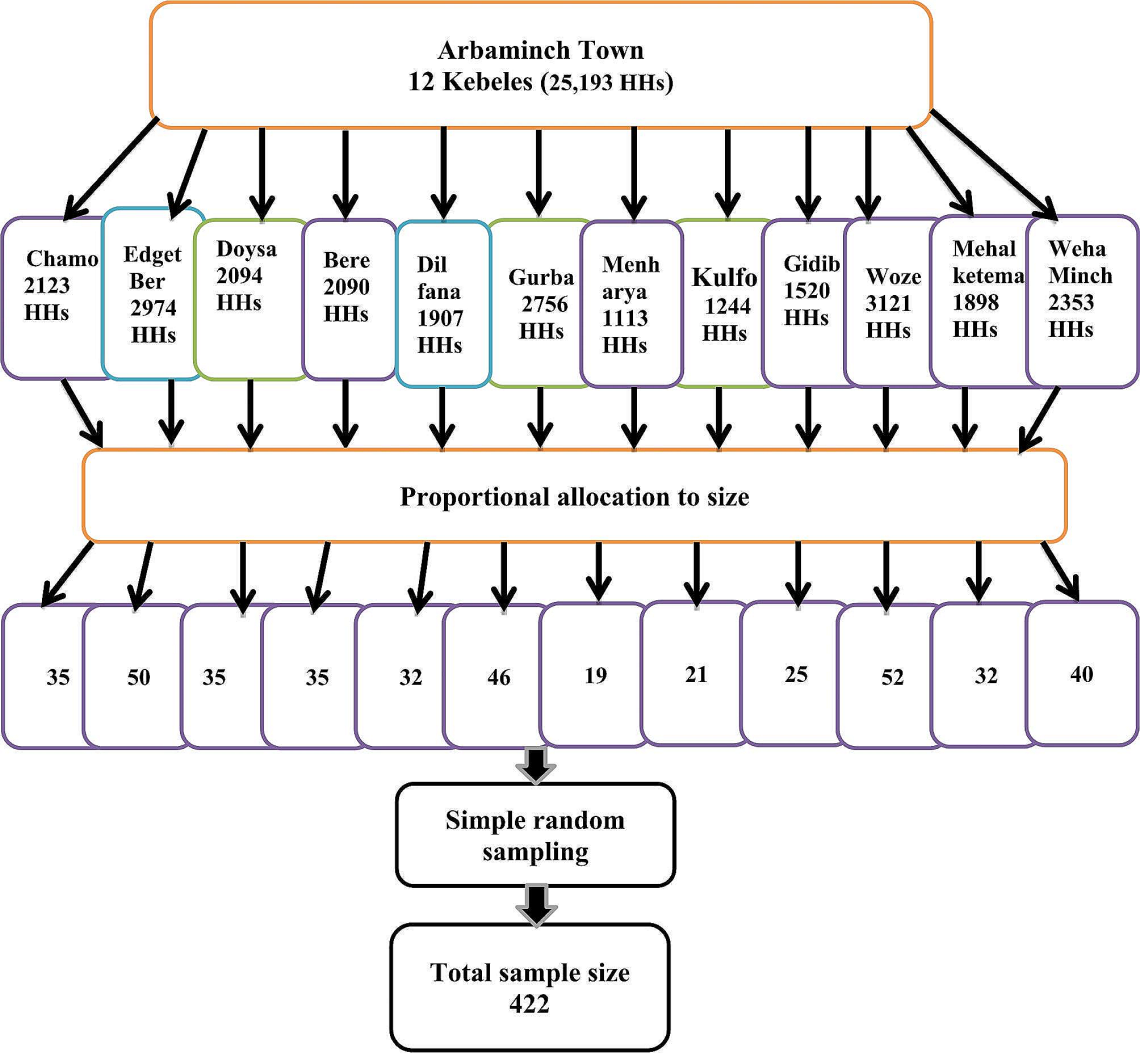
Schematic presentation of the sampling procedure for a study on health care seeking behavior towards cervical cancer among 30–49 aged women in Arba Minch town, Southern Ethiopia, 2023

Simple random sampling method was employed to select the study subjects. There are a total of twelve kebeles in Arba Minch town. List of households with women of 30–49 age were obtained from health extension workers of the respective kebeles. Samples were allocated to each kebele proportionally to their size. Then, the sample was drawn using a simple random sampling technique via computer-generated random numbers. Randomly selected households with eligible women were traced using their house numbers and health extension workers were used as guidance. Lottery method was used when more than one eligible woman were encountered in the household. (Fig. 2)

### Data collection and measurement

The data was collected by face-to-face interview using pretested structured questionnaires using the kobo tool box mobile application. The questionnaire was prepared in English and then translated into Amharic and back to English for checking language consistency by different person with an excellent metalinguistic skill who can understand the context. Two days of training was given for two BSc midwifes. Before data collection, pre-test was conducted on 5% of the computed sample at Chencha town. Depending on the feedback obtained from the pretest result, necessary correction and arrangement of sensitive questions were made to improve the clarity, understandability, and simplicity. The collected data was checked for completeness and consistency by the investigator. Using Cronbach’s alpha, the reliability and internal consistency of the HBM constructs were calculated independently. Perceived susceptibility was measured by 3 items with Cronbach alpha of 0.81, perceived severity was measured with 6 items with Cronbach alpha of 0.83, while perceived benefit was measured with 4 items which gave Cronbach alpha of 0.84 and perceived barriers were measured with 6 items with Cronbach alpha of 0.89. The collected data was checked for completeness and consistency by the investigator.

The questionnaire was developed based on the modified and adapted Champion’s health belief model scales [14, 15] and review of other literatures [16–21]. The questionnaires have the following parts: The socio-demographic characteristics consisted of age, occupation, educational background, religion, marital status, and monthly income.

- **Knowledge about cervical cancer and screening**: A total of 11 items were used to assess the participant’s knowledge and correct answers were categorized as 1 and incorrect answers were categorized as 0. The maximum point value was 11 and the minimum point was 0. Then, the respondent’s knowledge was categorized either in to good knowledge (those who scored above the mean) and poor knowledge (those who scored below the mean) based on the cumulative mean score of participants’ knowledge on cervical cancer [18].
- **Perceived severity of cervical cancer**: Women’s beliefs about the seriousness and complications of cervical cancer [15, 21].
- **Perceived susceptibility for cervical cancer**: Perception of the woman about the chances of experiencing a risk or getting cervical cancer [19, 21].
- **The perceived benefit of undergoing cervical cancer screening**: Women’s perception regarding the efficacy of undergoing cervical cancer screening to reduce the risk or seriousness of cervical cancer [8, 15, 21].
- **Perceived barriers for undergoing cervical cancer screening**: Beliefs about the tangible and psychological costs of obtaining cervical cancer screening [15, 21].
- **Perceived self-efficacy**: Women’s perception on her confidence to undergo cervical cancer screening.

**Cues to action**: Strategy to activate the decision-making process to get screened for cervical cancer and was measured using 3 items and participants who scored mean and above were considered as having positive cues to action [15].

Perceived severity, susceptibility, benefit, barrier, and self-efficacy were assessed using the Likert scale (1 = strongly disagree, 2 = Disagree, 3 = Neutral, 4 = Agree, 5 = strongly agree). Mean scores were computed for each construct and dichotomized into high/positive and low/negative [14, 15, 22].

**Health care seeking behavior**: it was measured using four items with five points of Likert scale and based on the mean score; women were classified in to having health seeking behavior and not having health seeking behavior [7].

### Data processing and analysis

The collected data was first cleaned and exported from kobo collect to SPSS Version 27.0 for further management and analysis. Descriptive statistics was computed and described using tables, figures, and charts. Binary logistic regression analysis was used to see the independent effect of predictors on health care seeking behavior. Those values with a *P* value < 0.25 during Bivariable analysis were retained for multivariable analysis. Odds ratio (ORs) with 95% corresponding confidence intervals (CIs) was calculated to measure the strength of the association between explanatory variables and the outcome variable. The statistical significance was considered at *P*-value less than 5%. Hosmer and Lemeshow model fitness test was used to test the model fitness and the value was 0.6. Multicollinearity was determined by variable inflation factor and no multicollinearity was found among the constructs of the HBM. Finally, the findings were presented using texts, tables, graphs, and charts.

## Results

### Socio demographic characteristics of participants

A total of 414 women participated in the study, giving a response rate of 98%. Majority 298 (72%) of the study participants were between the age categories of 30–39. The mean and standard deviation of the age of the study participants were 37.6 ± 5.3 years. Majority 347(83.8%) of the respondents were married. Regarding the educational status of the study participants, majority 228(55.1%) of the study participants had no formal education and only 74(17.9%) of the respondents were college and above graduates. (Table 1)

**Table 1:** Socio-demographic characteristics of the study participants in Arbaminch town, southern Ethiopia, 2023

Variables (*n* = 414)		Frequency	Percent
Age	30–39	298	72%
	40–49	116	28%
Ethnicity	Gamo	195	47.1%
	Goffa	84	20.3%
	Amhara	23	5.6%
	Wolaita	56	13.5%
	Konso	38	9.2%
	Others	18	4.3%
Religion	Orthodox	182	44%
	Muslim	46	11.1%
	Protestant	136	32.9%
	Catholic	18	4.3%
Occupation	House wife	139	33.6%
	Merchant	130	31.4%
	Gov’t employee	69	16.7%
	Private employee	50	12.1%
	Student	26	6.3%
Marital status	Married	347	83.8%
	Widowed	24	5.8%
	Divorced	18	4.3%
	Single	25	6.1%
Educational status	No formal education	228	55.1%
	Primary school	52	12.6%
	Secondary school	60	14.5%
	College and above	74	17.9%

### Participants knowledge regarding cervical cancer and its prevention

In this study, it were found that the majority, 329(79.5%) of the study participants had heard about cervical cancer. Of those who had heard about cervical cancer, the majority, 171(43.3%) had heard about cervical cancer from health care providers. Sexually transmitted disease 105(25.4%), Smoking 47(11.4%), having multiple sexual 98(23.7%), Poor dietary habit 58(14%), Early marriage 32(7.7%), others 74(17.9%) were risk factors of cervical cancer mentioned by the respondents. (Table 2)

**Table 2:** Participants knowledge regarding cervical cancer in Arbaminch town, Southern Ethiopia, 2023 (*n* = 414)

Variables	Response	Frequency	Percentage
Heard about cancer	Yes	329	79.5%
	No	85	20.5%
Know the risk factors for cervical cancer	Yes	330	79.7%
	No	84	20.3%
Knowledge on risk factors of cervical cancer	Sexually transmitted disease	105	25.4%
	Smoking	47	11.4%
	Having multiple sexual partners	152	36.7%
	Poor dietary habit	58	14%
	Early marriage	32	7.7%
	Others	20	4.8%
HPV is a causative agent of cervical cancer	Yes	330	79.7%
	No	84	20.3%
Know common symptoms of cervical cancer	Yes	326	78.7%
	No	88	21.3%
Knowledge on the symptoms of cervical cancer	Intra or post coital bleeding	128	30.9%
	Bleeding after menopause	104	25.1%
	Persistent blood stained vaginal discharge	126	30.4%
	Lower abdominal pain	56	13.5%
Cervical cancer is preventable	Yes	329	79.5%
	No	85	20.5%
Cervical cancer is curable	Yes	332	80.2%
	No	82	19.8%
If yes, where did you hear about cervical cancer screening for the first time?	Relatives	163	39.4%
	Friends	70	16.3%
	Health workers	171	41.3%
	Mass media	10	2.4%
How many times should a healthy woman undergo pap smear test?	Only once	148	35.7%
	Two times only	133	32.1%
	At list three times and above	133	32.1%
Does anyone in your first-degree relatives have a history of cervical cancer?	Yes	46	11.1%
	No	368	88.9%

### Health care seeking behavior

**Fig. 3:**
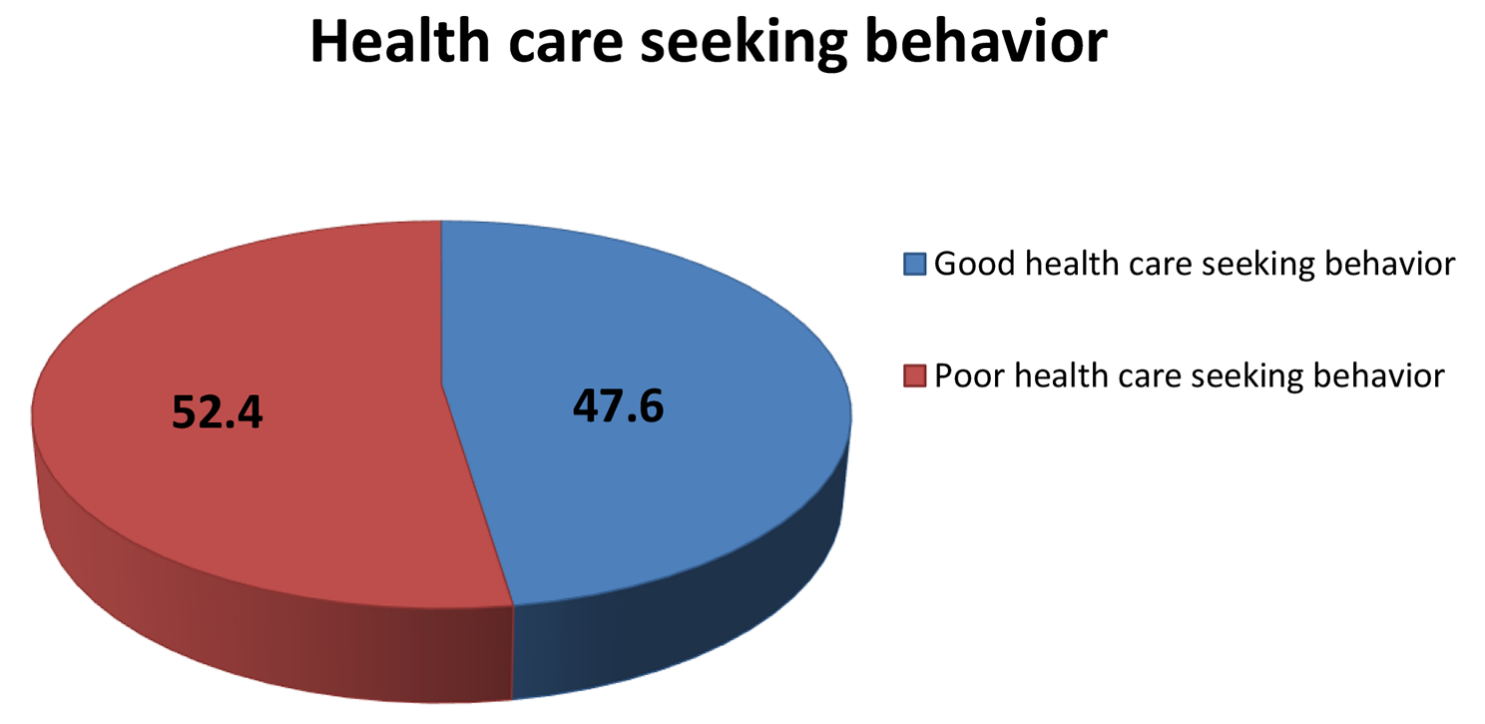
Health seeking behaviour of the study participants among 30–49 years women in Arba Minch town, southern Ethiopia, 2023

The proportion of health care seeking behavior towards cervical cancer screening among the respondents was 197(47.6%) [95%CI: 42.7-52.5%]. Of which, 178(90.4%) of the study participants had heard about cervical cancer screening. The information was provided for 81(41.1%) by Relatives, 78(39.6%) by health workers, 34(17.3%) by friends, and 4(2%) by Mass media respectively. (Fig. 3)

### Constructs of the health belief model

About 274(66.2%) of the study participants had a positive perceived benefit towards cervical cancer screening. Half 207(50%) of the respondents had a positive perceived severity towards cervical cancer screening. Only eighty-three (20%) of the respondents had a positive perceived susceptibility towards cervical cancer screening. (Table 3)

**Table 3:** Constructs of health belief model for the study of health seeking behavior towards cervical cancer screening and associated factors among 30-49years women in Arbaminch Town, southern Ethiopia, 2023

Items	Strongly disagree	Disagree	Neutral	Agree	Strongly agree	Mean (± SD)
Perceived benefit						
Cervical cancer screening contributes to early detection of cervical cancer.	9	26	9	86	284	4.47(0.97)
Cervical cancer screening reduces the risk of death from cervical cancer.	105	72	56	69	112	3.03(1.56)
Performing a Cervical cancer screening test will relieve my stress.	100	77	51	74	112	3.12(1.48)
Cervical cancer screening can lead to early detection of uterine infections.	102	80	50	69	118	3.14(0.98)
Perceived barriers						
I’m embarrassed to get a cervical cancer screening.	116	38	128	22	110	2.93(1.53)
I do not have enough time to go for cervical cancer screening.	39	112	17	83	163	3.53(1.47)
Cervical cancer screening is painful for me.	110	46	121	39	100	2.93(1.53)
I’m afraid of performing a cervical cancer screening because I do not know what it will be like.	114	36	120	20	108	2.93(1.53)
I do not go for the cervical cancer screening because I fear the result might be positive.	133	43	18	28	192	3.25(1.81)
I’m skeptical about the effectiveness of cervical cancer screening for preventing cervical cancer.	160	109	29	44	72	2.42(2.42)
Perceived self-efficacy						
I can easily perform cervical cancer screening.	265	117	6	22	4	1.51(0.85)
I can easily adjust my time to perform cervical cancer screening.	271	110	16	14	3	1.47(0.79)
I can easily overcome my fear of performing cervical cancer screening.	259	110	11	26	8	1.57(0.94)
I can easily overcome my embarrassment of performing cervical cancer screening.	105	72	56	69	112	3.03(1.56)
Perceived severity						
Cervical cancer is a dangerous disease.	82	75	50	65	142	3.27(1.56)
Cervical cancer is fatal.	9	26	9	86	284	4.47(0.97)
If someone suffers from cervical cancer, her sexual relationship will be affected.	61	75	71	65	142	3.27(1.56)
Even thinking about suffering from cervical cancer scares me.	80	72	49	63	140	3.27(1.56)
Cervical cancer leads to disease-related problems for a long time.	90	75	53	61	135	3.27(1.56)
Cervical cancer disrupts the person’s life.	8	69	48	63	139	3.27(1.56)
Perceived susceptibility						
There is a risk of cervical cancer for all women.	330	65	1	18	0	1.29(0.68)
The risk of cervical cancer is high in me in the next few years.	298	90	4	20	1	1.23(0.70)
There is a risk of cervical cancer at any age.	331	65	0	18	0	1.01(0.65)

### Factors associated with health care seeking behavior

In the current study, the respondent’s good knowledge, positive perceived susceptibility, positive perceived severity, and positive perceived benefit were factors associated with good health care seeking behavior towards cervical cancer screening, whereas variables like perceived self-efficacy, perceived barriers and cues to action were not significantly associated with health care seeking behaviour. (Table 4)

**Table 4:** Bivariate and Multivariable analysis of factors associated with health care seeking behavior towards cervical cancer screening among women aged 30–49 years and women in Arbaminch Town, southern Ethiopia, 2023

Variables		HSB		Crude OR (95% CI)	Adjusted OR (95% CI)	*P* value
		Yes	No			
Knowledge score	Good	121(61.4%)	118(54.4%)	1.34(0.90–1.98)	1.55(1.01–2.39)	0.032*
	Poor	76(38.6%)	99(45.6%)	1	1	
Perceived susceptibility	Positive	55(27.9%)	28(12.9%)	2.61(0.23–0.63)	3.63(2.06–6.42)	0.001*
	Negative	142(72.1%)	189(87.1%)	1	1	
Perceived benefits	Positive	156(79.2%)	118(54.4%)	3.19(0.20–0.48)	4.85(2.92–7.87)	0.001*
	Negative	41(20.8%)	99(45.6%)	1	1	
Perceived severity	Positive	118(59.9%)	89(41%)	2.15(1.45–3.18)	2.65(1.71–4.09)*	0.001
	Negative	79(40.1%)	128(59%)	1	1	
Cues to action	Positive	172(87.3%)	199(91.7%)	0.62(0.33–1.18)	0.84(0.39–1.79)	0.22
	Negative	25(12.7%)	18(8.3%)	1	1	
Perceived barriers	Positive	112(56.9%)	122(56.2%)	1.03(0.70–1.51)	1.08(0.69–1.68)	0.75
	Negative	85(43.1%)	95(43.8%)	1	1	
Perceived self-efficacy	Positive	108(54.8%)	114(52.5%)	1.10(0.75–1.61)	1.28(0.79–2.07)	0.31
	Negative	89(45.2%)	103(47.5%)	1	1	

*significantly associated factors, COR: Crude odds ratio, AOR: Adjusted odds ratio, 1 Reference group

## Discussion

This study determined the level of health care seeking behavior towards cervical cancer screening and associated factors among women in 30-49years. In the current study, 197(47.6%) [95%CI: 42.7-52.5%] of women had health care seeking behavior towards cervical cancer screening. This finding is higher than a study conducted in Hossana town, Southern Ethiopia, which showed only 14.2% of the participants had health care seeking behavior towards cervical cancer screening. This might be due to differences in the tools used in the current study and the increased level of awareness of the women about cervical cancer screening from time to time [7]. The present study is also higher than the study conducted in Nepal which showed only 18.3% of the participants had health seeking behavior towards cervical cancer screening. This could be due to the difference in the sample size and population characteristics of the two countries [10]. However, the current finding is lower than the previous study conducted in Arbaminch town on the utilization of cervical cancer screening which showed 71.5% of the study participants had an intention for cervical cancer screening [23]. In the current study, those participants who had good knowledge towards cervical cancer screening were 1.55 times more likely to have good health care seeking behavior towards cervical cancer screening than their counter parts (AOR = 1.55, 95%CI = 1.01–2.39). The finding of this study is consistent with studies conducted in Kenya, Johannesburg, Mekelle, and Jimma, Ethiopia [20, 24]– [26]. This might be due to the different activities that have been undertaken to increase the utilization of cervical cancer screening. For instance, health education by health care providers, campaigns prepared by health care providers and university students during team training programs about cervical cancer and its preventive methods, which in turn will result in modified attitudes and changed behavior.

Regarding the perceived susceptibility of women towards cervical cancer screening, this study revealed that those women who had positive perceived susceptibility were 3.63 more likely to have health care seeking behavior towards cervical cancer screening than their counter parts (AOR = 3.63, 95%CI = 2.06–6.42). This finding is in line with the studies conducted in Jimma and Mekelle Ethiopia [19, 25]. This might be due to these women who have had awareness about cervical cancer and perceived as they are at risk of getting cervical cancer are more likely to undergo cervical cancer screening to protect themselves.

Perceived benefits are the positive outcomes a woman believes will result if they decide to take action to reduce and/or prevent cervical cancer. This study revealed that those women who had positive perceived benefit towards cervical cancer screening were 4.85 times more likely to have health care seeking behavior towards cervical cancer screening than their counter parts (AOR = 4.85, 95%CI = 2.92–7.87). This finding is supported by study conducted in Bishoftu [17]. This might be due to an increase in the level of awareness of the benefits of cervical screening among women from time to time. However, studies conducted in Botswana, Nepal, and Jimma revealed that cervical cancer screening behavior of the respondents was independent of the perceived benefits of cervical cancer [10, 19, 21].

The result shows that study participants who had positive perceived severity were 2.65 times more likely to have health care seeking behavior than those women with negative perceived severity towards cervical cancer screening (AOR = 2.65, 95%CI = 1.71–4.09). This finding is supported by studies conducted in Ghana and Johannesburg which demonstrated a significant and positive correlation between perceived severity and screening behavior [20, 27]. However, a study conducted in Jimma reported that perceived severity was not significantly associated with cervical cancer screening. This might be due to previous traumatic experiences of women regarding cervical cancer or knowing someone who is suffering from the disease.

This study also revealed that there is no association between perceived self-efficacy of women towards cervical cancer screening and health care seeking behavior. Although it is generally stated by different studies and self-efficacy theory of Albert Bandura, about the relationship between positive self-efficacy and the likelihood of behavioral change, this study is in conflict with self-efficacy theory [28]. This might be due to the difference in the knowledge level of the women, lack of experience of cervical cancer, and low level of health seeking behavior of the women towards cervical cancer screening.

In the current study, no significant association was found between health care seeking behaviour towards cervical cancer screening and perceived barriers of cervical cancer screening which is in accordance with a study conducted in Ugrachandi Nala, Kavre, Nepal which revealed no significant association between perceived barriers and cervical cancer screening behaviour [10]. The possible explanation might be due to the high perceived benefits of the women, which might outwait the perceived barriers towards cervical cancer screening. The more women perceive the benefits of performing cervical cancer, the more she will bypass the barriers, which will prevent her from getting screening services. However, a study conducted in Latin America showed the association between seeking health care and perceived barriers of cervical cancer screening [29]. This might be due to the fear of the majority of the women about the test results and the thought that the screening procedure might be painful. Since most women feel uncomfortable with the idea of vaginal examination or ‘private parts, embracement might be another possible explanation.

### Limitations of the study

Since an interviewer-administered questionnaire was used, there was a possibility of validating what the participant responded, which has the potential to introduce a social desirability bias. Furthermore, because of the nature of the cross-sectional study design, it could be challenging to determine whether outcome or predictor variables come first. The drawback of the health belief model is also a limitation of this study. The study does, however, have certain strengths. The health belief model, which has helped to examine the woman’s beliefs in to four categories; perceived benefit, perceived susceptibility, perceived severity, and perceived barriers. This in-depth approach examines a woman’s beliefs regarding health care seeking behaviour in a more holistic way than any other model.

## Conclusion

The prevalence of health care seeking behaviour towards cervical cancer screening was low in the study area. Respondents’ good knowledge, positive perceived susceptibility, positive perceived severity, and positive perceived benefits were significantly associated with health seeking behavior.

Women’s health care seeking behavior towards cervical cancer screening is maximized by acting on the degree to which the woman feels susceptible to cervical cancer, knowledge regarding cervical cancer and its screening, the degree to which women believe the consequences of cervical cancer will be severe, the benefit the woman will get from cervical cancer screening.

## Data Availability

The corresponding author will provide the datasets used and/or analyzed during the current study upon reasonable request.

## Abbreviations

AOR: Adjusted odds ratio
CI: Confidence Interval
COR: Crude odds ratio
HBM: Health Believe Model
HPV: Human papilloma virus
